# Efficacy and safety of various drug combinations in treating plaque Psoriasis: A meta-analysis

**DOI:** 10.12688/f1000research.149172.1

**Published:** 2024-05-07

**Authors:** Nayak Snehasis, Sayed Zafar, Ngabo Herve, Pendyala Siri, Karshe Haji Ali

**Affiliations:** 1 Medicine, University of Visayas Gullas college of Medicine, Banilad, Cebu, 6000, Philippines

**Keywords:** PASI 75%, RoB2, Pioglitazone, Combination therapy

## Abstract

**Background:**

Psoriasis, a chronic inflammatory disease affecting the skin, joints, and nails (2-3% worldwide), significantly affects quality of life. Genetic and environmental factors also play key roles. Topical corticosteroids, calcineurin inhibitors, and oral corticosteroids help to manage plaque psoriasis symptoms, but combination therapies might offer greater effectiveness and improved safety profiles. These combinations could potentially reduce medication dosages and side effects in patients.

**Objective:**

To assess the efficacy and safety of various drug combinations over conventional monotherapies in the treatment of moderate to severe plaque psoriasis, which incorporates palmoplantar psoriasis and psoriasis vulgaris, by discovering and utilizing research articles comprising the same or similar variables as PASI 75 and evaluating the information using RevMan v5.4.1.

**Method:**

We reviewed the efficacy and safety of combination therapy vs. monotherapy/placebo for moderate-to-severe plaque psoriasis (palmoplantar and vulgaris) using PASI 75 via RevMan v5.4.1. Risk of bias assessment and funnel plots were employed to assess the heterogeneity of each paper. Inclusion criteria – Publications < 20yrs, RCTs, plaque/palmoplantar/psoriasis vulgaris; exclusion criteria – Guttate/arthritic psoriasis, pediatric and pregnant individuals, publications > 20 yrs.

**Results:**

Seventeen studies were analyzed, comprising a total of 2291 patients (n=1147 with combination regimens and n=1144 with control regimen in the analysis). A significant PASI 75- response was observed in the pioglitazone combination subgroup as compared to placebo (OR=4.92,95% CI 2.19-11.05, P = 0.0001); methotrexate combination subgroup as compared to placebo (OR=2.56, 95% CI 1.67-3.94, P< 0.0001) test of subgroup differences showed P= 0.14, I ^2^= 34%. Incidence rate of abnormality in levels of liver enzymes (OR=1.89,9.5% CI 6.69-5.22, P=0.22), nausea (OR=1.28,95% CI 0.77-2.14, P=0.34), headache (OR=1.28,95% CI 0.77-2.14, P=0.58), fatigue (OR=0.89, 95% CI 0.41-1.90, P=0.45).]

**Conclusion:**

This study showed that combination therapy is very effective for plaque psoriasis, with promising combinations of pioglitazone. While safety seems similar between the groups, larger studies are needed to determine the long-term effects. These findings suggest that personalized treatment plans could improve outcomes; however, confirmation through larger trials is crucial before wider use. This opens doors to research on optimal combinations for individual patients.

## 1. Introduction

Psoriasis is a chronic, inflammatory, and multisystemic disease that can cause considerable morbidity and adversely affect quality of life. ^1^^,^^2^ It affects 2-3% of the global population and is commonly seen in genetically predisposed individuals triggered by environmental factors that mainly affect the skin, nails, and joints. ^3^

The precise cause of plaque psoriasis is unknown; however, it is thought to be a combination of hereditary and environmental factors. The immune system incorrectly assesses healthy skin cells, resulting in an overabundance of skin cells and inflammation, which results in distinctive plaques.

Plaque psoriasis pathogenesis involves a complicated interplay between the immune system, genetics, and environmental variables. The immune system plays an important role in plaque psoriasis development. T cells, which are white blood cells, are activated and concentrated in the skin causing inflammation. ^4^

Interleukin-17 (IL-17), tumor necrosis factor-alpha (TNF-alpha), and interleukin-23 (IL-23) are cytokines that play a role in the inflammatory response. These cytokines increase keratinocyte proliferation and differentiation, resulting in psoriatic plaque production. ^5^ Genetics also plays a role in the development of plaque psoriasis. Many genes have been found to contribute to the development of the disease, including HLA-Cw6, which has been linked to an elevated risk of psoriasis. Other immune response genes such as interleukin-12 (IL-12) and Interleukin-23 (IL-23) have also been implicated. ^6^^,^^7^

Environmental variables, such as stress, infection, and drugs, can cause or worsen plaque psoriasis. Streptococcal infections have been linked to the onset of psoriasis in some patients. Lithium, beta-blockers, and antimalarial have also been associated with the development of psoriasis. ^8^

Current monotherapy options for moderate-to-severe plaque psoriasis often have limitations, including partial response, safety concerns, and the development of resistance. ^8^ Combination therapy for plaque psoriasis has emerged as a promising approach to overcome the limitations of monotherapy, aiming to improve efficacy, reduce side effects, and potentially delay resistance. ^9^

In this study, we intended to compare the efficacy utilizing PASI 75 score which is a binary outcome that indicates 75% or greater improvement in PASI from baseline ^25^ and safety of some of the drug combinations statistically used in the treatment of disease-focused (i.e., plaque psoriasis, psoriasis vulgaris, palmoplantar psoriasis) using a few manuscripts in the databases that talked about random trial techniques where the drug combinations were compared with the results of placebo; thus, we used the software, RevMan v5.4.1, a freely available software, initially built to ease the planning of protocols, the writing of reviews, and the storing of information within the Cochrane organization.

Reviews can be systematic or nonsystematic. In our systematic review, we aimed to incorporate quantitative analysis method, such as meta-analysis; RevMan v5.4.1 *,* which helped us do the job and additionally produced some interesting graphical outputs of the data entered. It offered a variety of statistical features and methods, such as effect size calculations, heterogeneity tests, subgroup analyses, risk of bias summary and risk of bias graph, and publication bias *.* This enabled us to synthesize data from various studies, visualize data using forest and funnel plots, and produce more accurate and reliable estimations of treatment effects.

Reducing inflammation, itchiness, and redness is the main goal of psoriasis treatment. ^10^ Classes of drugs, such as topical corticosteroids, topical retinoids, topical calcineurin inhibitors, and oral medications, such as methotrexate, cyclosporine, and acitretin, are being used. ^11^^,^^12^

Although several systematic reviews provide a summary of studies that report combination therapy with systemic agents, ^9^^,^^13^^,^^14^ risk of bias assessments were only found in one throughout our search. ^1^

In this study, we combined drugs such as pioglitazone, methotrexate, apremilast, etanercept, calcipotriol, betamethasone, cyclosporine, clobetasol propionate, acitretin, fumarate, and cetirizine using RevMan v5.4.1, additionally Forest plots were used for better visualization and analysis of the efficacy and safety results by comparing the odds ratio. For papers with graphical representations, ^15^ extra software such as web plot digitizer and Python plot were used to extract PASI 75 values.

## 2. Method

### 2.1 Data search and extraction

We performed a systematic literature study using Mendeley software to identify randomized controlled trials that compared the efficacy and safety of different drug combinations for plaque psoriasis and searched several stand-alone databases, including Google Scholar, PubMed, Pub Chem, Wiley library, MEDLINE, JDD Library, and the Cochrane Library. Considering the rapid evolution of knowledge and methodological standards in psoriasis research, particularly over the past two decades, we chose to limit our search to studies published after 2004. This ensures that our analysis reflects the most recent and methodologically sound evidence relevant to the current clinical practice.

Treatment regimens between the time period of 8 and 24 weeks were chosen based on the understanding that studies conducted over shorter durations (<8 weeks) may not capture the full spectrum of treatment effects, potentially leading to biased conclusions. Conversely, long-term studies (>24 weeks) may introduce confounding variables or dropout rates, thereby compromising the reliability of the findings. Additionally, by encompassing a moderate timeframe, research within the 8–24-week range enables a balanced evaluation of both short-term and intermediate-term outcomes. This timeframe minimizes bias and ensures a comprehensive understanding of the drug’s efficacy and safety profile over a meaningful treatment period.

Some of the keywords included meta-analysis, safety, PASI 75, (psoriasis OR plaque psoriasis) AND (combination therapy OR drug combination OR poly-therapy) AND (randomized controlled trial OR clinical trial OR randomized trial).

As per the protocol, the reference lists of relevant articles and their reviews were thoroughly analysed and shortlisted for additional studies. Duplicates were eliminated using the Mendeley method.

Desktop v1.19.8 and screening the titles and abstracts of the remaining records for eligibility.

The PICO framework was used to formulate the research question and define the inclusion and exclusion criteria for the studies. The PICO elements were:

• Population: Patients with moderate to severe plaque psoriasis/Palmo-plantar and psoriatic vulgaris
• Intervention: Drug combinations
• Comparison: Placebo or monotherapy
• Outcome: The proportion of patients who achieved at least 75% improvement in the Psoriasis Area and Severity Index (PASI 75) and incidence of adverse events.

### 2.2 Inclusion criteria

The years of publication were less than 20 years. Plaque Psoriasis (which also includes Palmo-planter psoriasis and psoriasis vulgaris), randomized controlled trials, parallel group, 8-24 weeks treatment regimes, and combination drugs. Articles written in English. Body surface involvement was ≥5%. We included studies that reported the proportion of patients who achieved at least 75% improvement in the Psoriasis Area and Severity Index (PASI 75) and the incidence of adverse events; studies with comparable baseline characteristics were included. ^16^

### 2.3 Exclusion criteria

Abstract/title with psoriatic arthritis, guttate arthritis, erythrodermic psoriasis, non-randomized controlled trial papers (observational studies, retrospective studies, case reports, and review articles), >24 weeks treatment regimes, involvement of paediatric patients, non-English articles, studies that did not compare drug combinations with placebo or monotherapy.

We excluded studies that did not report the proportion of patients who achieved at least 75% improvement in the Psoriasis Area and Severity Index (PASI 75), studies without comparable baseline characteristics, large sample sizes leading to heterogeneity, and trials that included patients with ongoing previous treatment regimens.

Indirect comparisons were performed to compare the efficacy and safety of different drug combinations for plaque psoriasis because there were insufficient trials that directly compared them with placebo. We used trials that evaluated the combination of drugs A and B versus drug B monotherapy as a surrogate for drug A combination versus placebo. The following assumptions were made for indirect comparisons:

(1) The effect of the drug A and drug B combination was equal to the sum of the effects of drugs A and B alone.
(2) The effect of drug B monotherapy was comparable to that of placebo.
(3) The effect of drug A was homogeneous across different combinations.

Five reviewers independently extracted data from the included studies using a standardized form. We extracted the following information: study characteristics (authors, year, country, design, duration, and sample size), intervention details (drugs, doses, frequency, and route of administration), and outcome measures (PASI 75, adverse events). The Cochrane risk of bias tool (RoB 2) was used to evaluate the methodological quality of the studies. Any discrepancies were resolved by discussion or consultation with other reviewers. We mailed the authors of the original studies for missing or unclear data.

We used WebPlotDigitizer v4.7 and Python Plotly v5.18.0 to extract the PASI 75 data from the Morita paper, which did not report the exact data numbers. We calculated the odds ratio (OR) and 95% confidence interval (CI) for each study by using the extracted data. We performed a meta-analysis using a random-effects model to estimate the pooled OR and 95% CI for the comparison of drug combinations with placebo or monotherapy. We assessed heterogeneity across studies using the I ^2^ statistic and the chi-square test.

We explored the potential sources of heterogeneity using subgroup analyses, based on the following variables: disease severity, including indices such as PASI 75, and risk of bias inherent in the studies under review. To evaluate the safety profile of the medications, we gathered data on adverse events (AE), including the prevalence of common side effects, such as abnormal liver enzymes, nausea, headache, and fatigue among the patient cohorts.

Publication bias was assessed using funnel plots. RevMan v5.4.1 , was used for all statistical analyses. We followed the PRISMA guidelines for reporting systematic reviews and meta-analyses by creating the PRISMA checklist.

## 3. Results

### 3.1 Literature results

**Figure 1.**
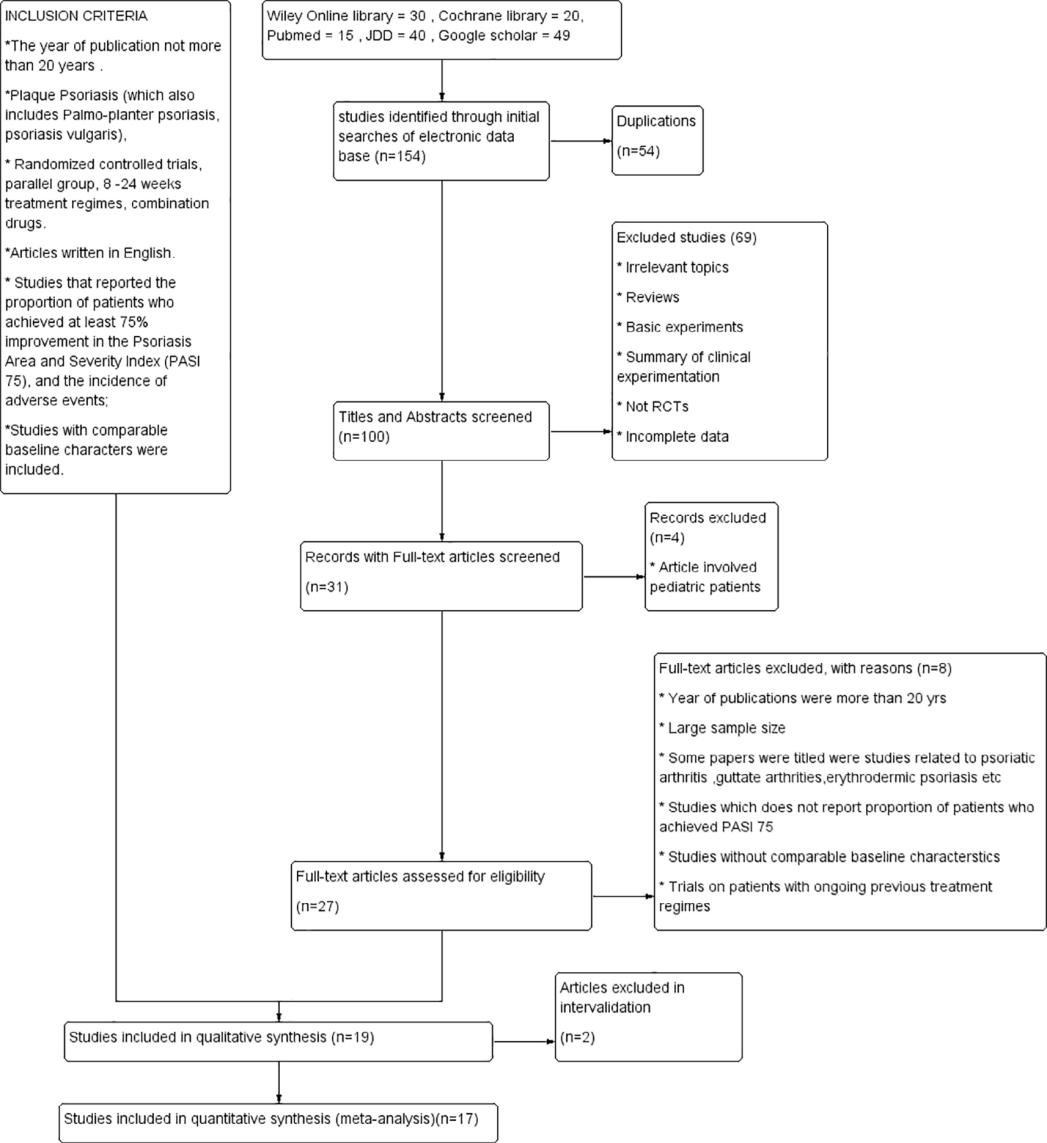
PRISMA flowchart.

A total of 154 records were initially identified through a comprehensive search across the following databases: PubMed (n = 15), JDD (n = 40), Cochrane Library (n = 20), Wiley Online Library (n = 30), and Google Scholar (n = 49). After removal of duplicates, 100 unique records were screened, leading to the exclusion of 69 articles for reasons such as not being randomized controlled trials (RCTs), didn’t utilize PASI as a way to measure efficacy, ^17^^,^^18^ lacking relevance to psoriasis, or focusing on drugs not covered in this review. Subsequently, 27 articles were included in the full-text review, of which 17 met our inclusion criteria and were consequently incorporated into this meta-analysis (refer Figure 1). The details of the materials are listed in Table 1.

**Table 1.** Baseline characteristics of included studies.

S.NO	Author Name	Year Publicaation	Type of Study	Drug	Time Period	Sample Size	Exp/Con(n)		Gender (m/f)		Age,y		Baseline PASI	
											Exp	Con	Exp	Con
**1**	Ghiasi	2018	Combination	Pioglitazone	10	60	30	30	18/12	19/11	42.8(15)	44.0(16.9)	20.9(9.8)	22.0(8.5)
**2**	Gisondi	2008a	Combination	Acitretin	24	40	18	22	9/9	12/10	53.4(12.3)	55.3(10.9)	11.9(6.5)	11.0(4.5)
**3**	Gisondi	2008b	Combination	Etanercept	24	38	18	20	9/9	12/8	53.4(12.3)	55.0(11.3)	11.9(6.5)	10.4(5.3)
**4**	Hassanandani	2023	Combination	Aprimilast	16	60	30	30	17/13	16/14	40.87(10.57)	44.47(9.91	21.73(2.70)	20.83(2.4)
**5**	Lejavardi	2015a	Combination	Pioglitazone	10	44	22	22	19/3	20/2	36.18(12.79)	42.5(16.09)	17.63(6.99)	18.61(8)
**6**	Lebwohl	2013	Combination	Clobetasol Propionate	12	592	295	297	195/100	196/101	43.7(14.2)	44.6(13.7)	18.9(9.2)	18.2(8.3)
**7**	Mittal	2009	Combination	Pioglitazine	12	41	19	22	19/0	21/1	42.2(10.6)	38.1(11.5)	17.5(4.6)	19.7(5.8)
**8**	Morita	2022	Combination	Aprimilast	8	40	27	13	18/9	10/3	38.77(15.03)	65.7(14.8)	12.4(8.47)	17.08(11.14)
**9**	Singh	2021	Combination	Cyclosporine	12	140	70	70	51/19	51/19	38.77(15.03)	38.04(14.97)	20.1(10.33)	18.26(9.65)
**10**	Vena	2012	Combination	Calcipotriol/Betametazone	8	60	30	30	24/6	22/8	49(9.33)	50.5(7.5)	23.7(12.9)	25.1(12.9)
**11**	Abidi	2020a	Combination	Pioglitazone	12	59	30	29	No Data	No Data	No Data	No Data	18.12(1.419)	17.68(1.1103)
**12**	Abidi	2020b	Combination	Methotrexate	12	60	30	30	No Data	No Data	No Data	No Data	18.12(1.49)	17.73(1.203)
**13**	Gottlieb	2012	Combination	Methotroxate	24	478	239	239	153/86	167/72	43(13.1)	45.2(12.8)	18.2(8.2)	18.3(6.6)
**14**	Liu	2019	Combination	Methotroxate	24	455	226	229	180/46	175/54	43.86(12.93)	42.31(11.75	24.27(9.78)	26.22(12.13)
**15**	Mahajan	2010	Combination	Methotrexate	12	40	20	20	17/3	12/8	36.90(11.48)	37.30(10.94)	16.02(3.51)	14.44(2.80)
**16**	Bezoojen	2016	Combination	Fumarate	24	33	18	15	14/4	8/7	43(17)	45(16)	12(10-16)	14(11-21)
**17**	Balak	2015	Combination	Cetirizine	12	50	25	25	15/10	18/7	46(31-62)	38(30-54)	2.7(10.8-16)	14.5(12.0-16.7)

### 3.2 Risk of bias

**Figure 2.**
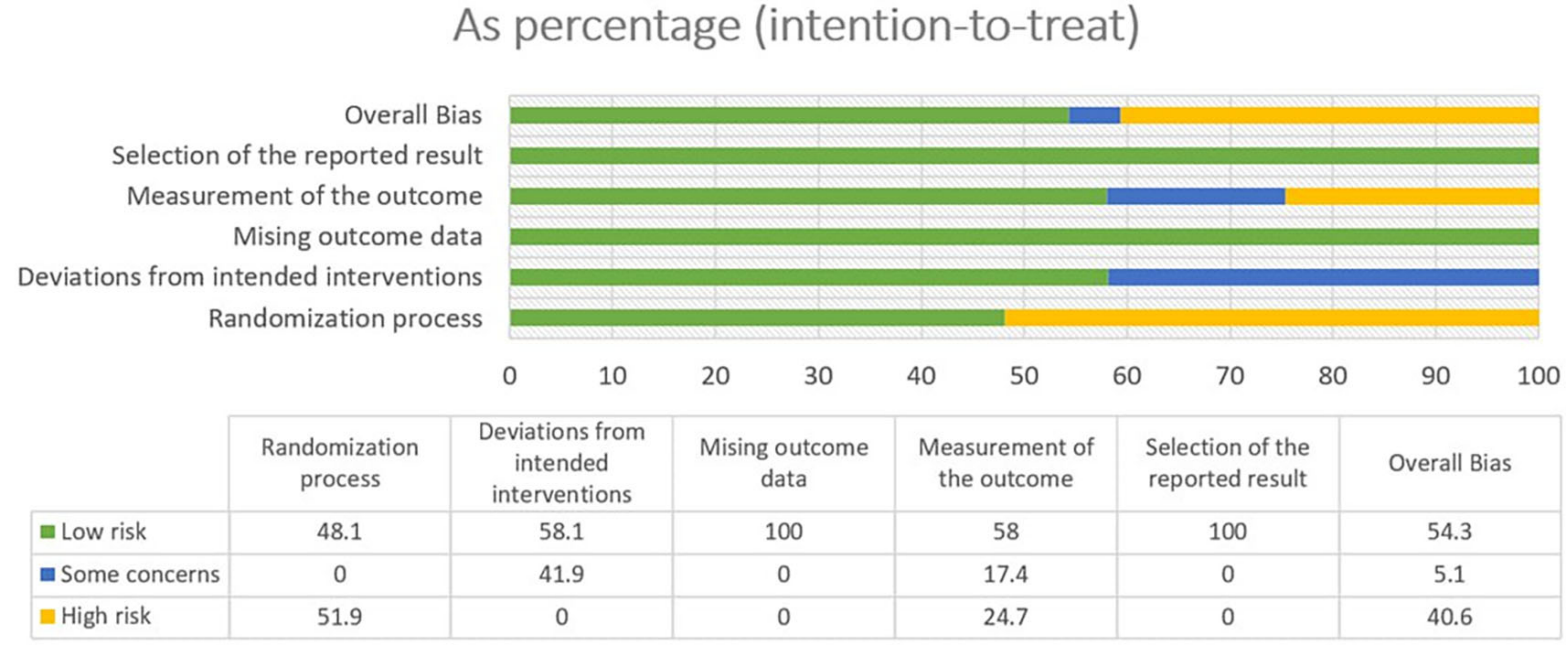
Risk of Bias 2 graph of ITT studies.

**Figure 3.**
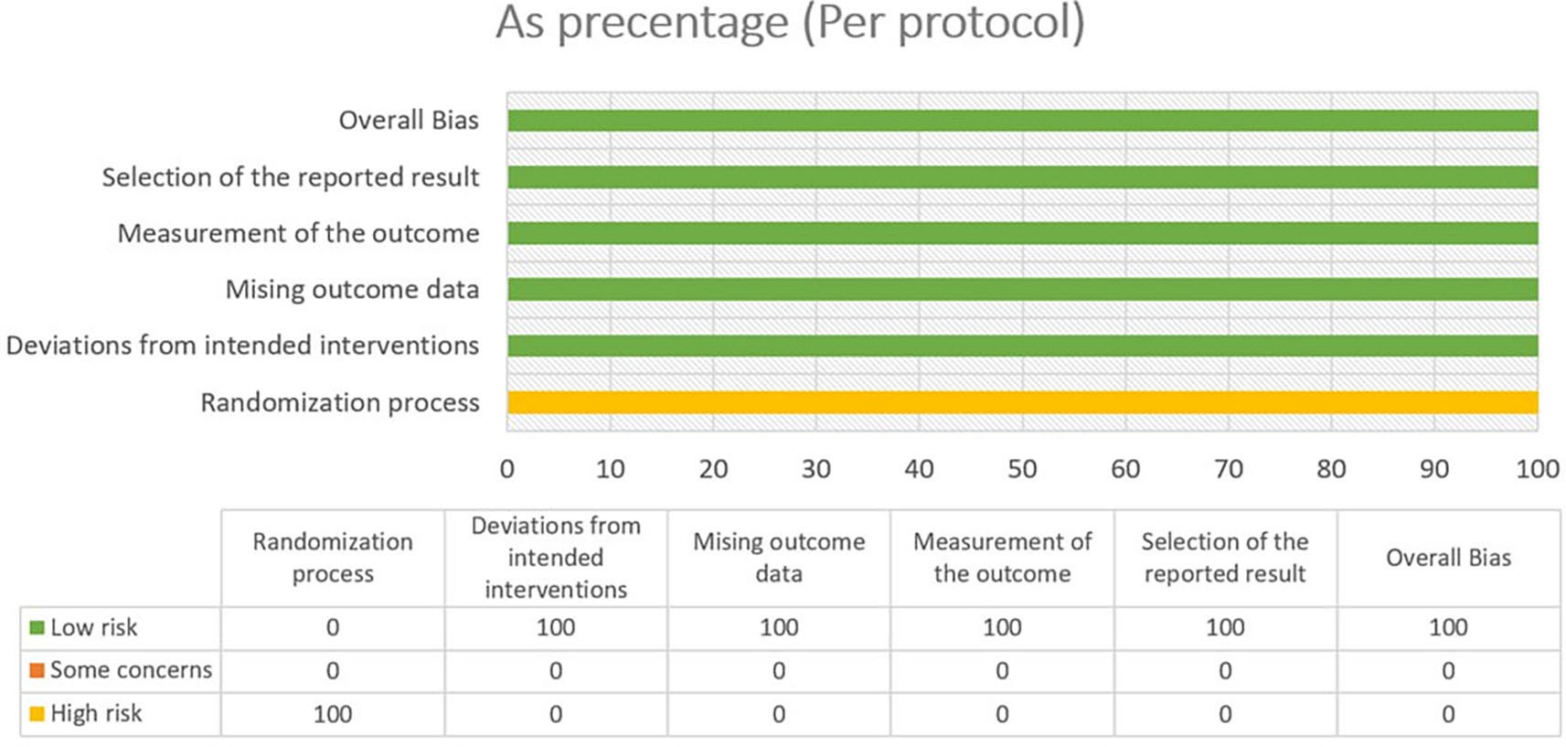
Risk of Bias 2 graph of PPS study.

**Figure 4.**
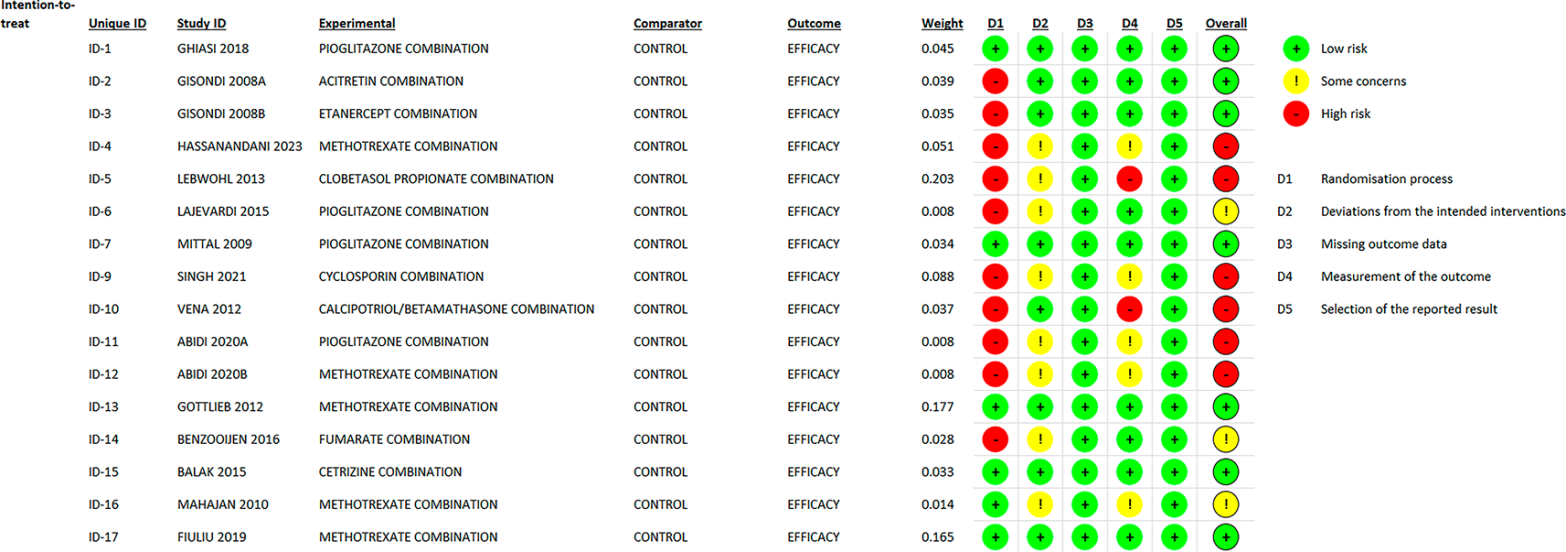
Risk of Bias 2 Summary of ITT study.

**Figure 5.**
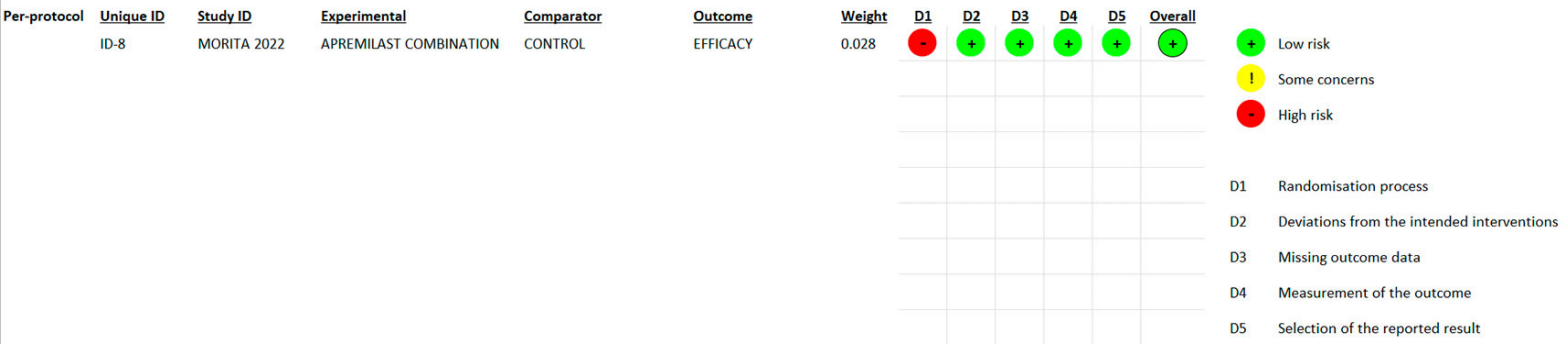
Risk of Bias 2 summary of PPS study.

In most areas, most ITT trials had a low risk of bias, indicating a high level of scientific rigor. Nonetheless, a few issues were observed in terms of deviations from the intended interventions, indicating potential areas for future investigations. The fact that no domain was predominantly related to a considerable risk of bias demonstrated the overall reliability of the included RCTs. Furthermore, one study that used PPS reported a low likelihood of bias. Because of the prevalence of open-label and single-blinded trials, the randomization domain may be more prone to bias than the others. However, because meta-analyses are intrinsically heterogeneous, ^19^ they emphasize the importance of carefully assessing diversity because they do not automatically imply biased conclusions (refer Figures 2, 3, 4 and 5).

### 3.3 Funnel plot interpretation

**Figure 6.**
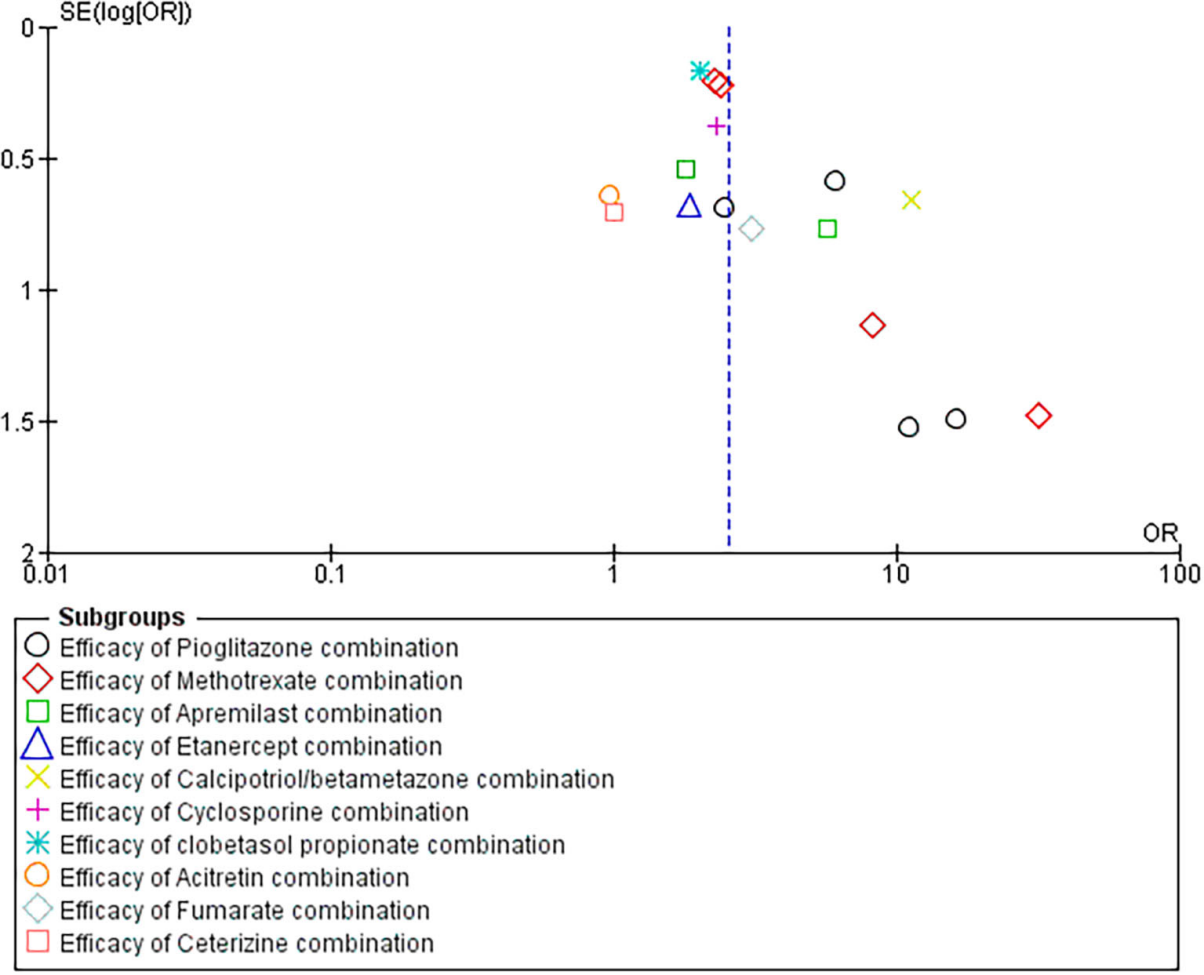
Funnel Plot analysis for included studies.

Our funnel plot analysis revealed a symmetric distribution along the x-axis, indicating that the studies were evenly distributed across the range of standard errors (SE) of the logarithm of the odds ratio (log [OR]). However, upon closer examination, we found that the studies were grouped together and had higher SE (log [OR]) values. The observed clustering suggests heterogeneity among the included studies or variability due to differences in sample size (refer Figure 6).

### 3.4 Efficacy analysis

Our analysis used the Mantel-Haenszel random-effects model, which we considered well suited for achieving dichotomous outcomes, as well as better adjusting for study size, data stratification, and generalization across diverse populations. As is well known, the PASI 75 response was regularly used to assess the severity and prognosis of psoriasis patients, and it fulfilled therapeutic expectations in the vast majority of these cases. Therefore, PASI 75 was utilized in this meta-analysis to evaluate the therapeutic efficacy of pharmaceutical combinations.

**Figure 7.**
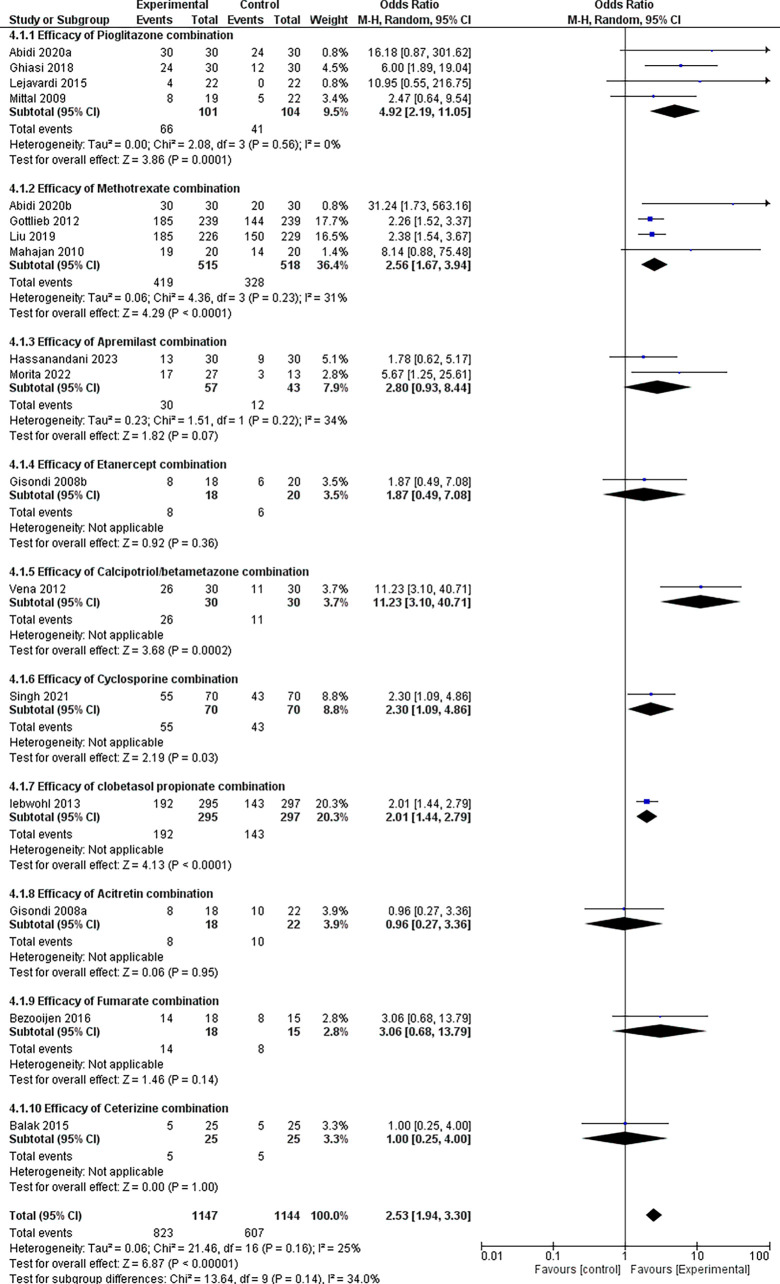
Forest Plot of efficacy of treatments in combination therapy verses Control therapy in patients with Plaque Psoriasis.

Our investigation found that combination therapy significantly increased the likelihood of positive outcomes by 2.53 times compared with controls (OR = 2.53, 95% CI: 1.94-3.30, p < 0.00001), refer Figure 7. The test for subgroup differences revealed no statistically significant variations in treatment effects (p = 0.14). However, the I-squared value (34%) indicated some heterogeneity across subgroups, necessitating further investigation using larger datasets.

Pioglitazone emerged as the frontrunner among the tested medication combinations, with an impressive odds ratio of 4.92 (95% CI: 2.19-11.05) and a very significant p-value of 0.0001. This study revealed a strong link between pioglitazone combination drugs and better outcomes compared with controls.

Several additional drug combinations showed promising results, with odds ratios of > 2 for methotrexate, cyclosporine, clobetasol propionate, and fumarate. These findings require further examination to confirm their potential benefits owing to data-size constraints.

Data availability for Acitretin and Cetirizine combinations was limited, with only a single study examining each. Although their respective odds ratios (0.98 and 1.00) did not reach statistical significance, drawing definitive conclusions based on single studies would be premature. This underscores the need for further research with larger sample sizes to assess their efficacy comprehensively.

Forest plots and subgroup analysis findings for the efficacy results are provided for easy reference.

### 3.5 Safety analysis

In our study exploring different drug combinations, we closely examined the side effects they might cause, such as abnormal liver enzymes, headaches, nausea, and fatigue.

**3.5.1 Abnormal liver enzyme findings**

**Figure 8.**
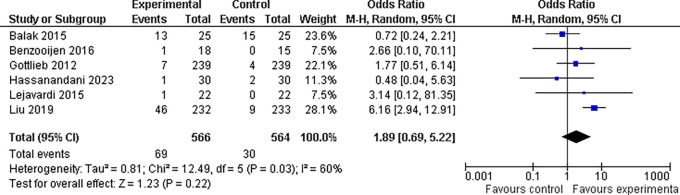
Forest Plot of Elevated/Abnormal liver enzymes in Combination Therapy verses control therapy patients with Plaque Psoriasis.

In our meta-analysis, we found that the overall odds ratio (OR) was 1.89 (95% CI: 0.69-5.22), indicating a nearly two-fold increase in risk; however, when we looked at the overall effect of the drug combinations on abnormal liver enzyme findings, the results were not statistically significant (P = 0.22). This suggests that based on the available evidence, we cannot definitively conclude that the drug combinations had a significant impact on abnormal liver enzyme readings. However, there was significant heterogeneity between trials (I ^2^ = 60%, P = 0.03), which could be related to variations in the study techniques, patient demographics, or treatment durations. It is also worth mentioning that ^20^ this study found the highest OR of 6.16, indicating a six-fold increase in ALE risk among patients receiving combination drugs (refer Figure 8).

**3.5.2 Nausea findings**

**Figure 9.**
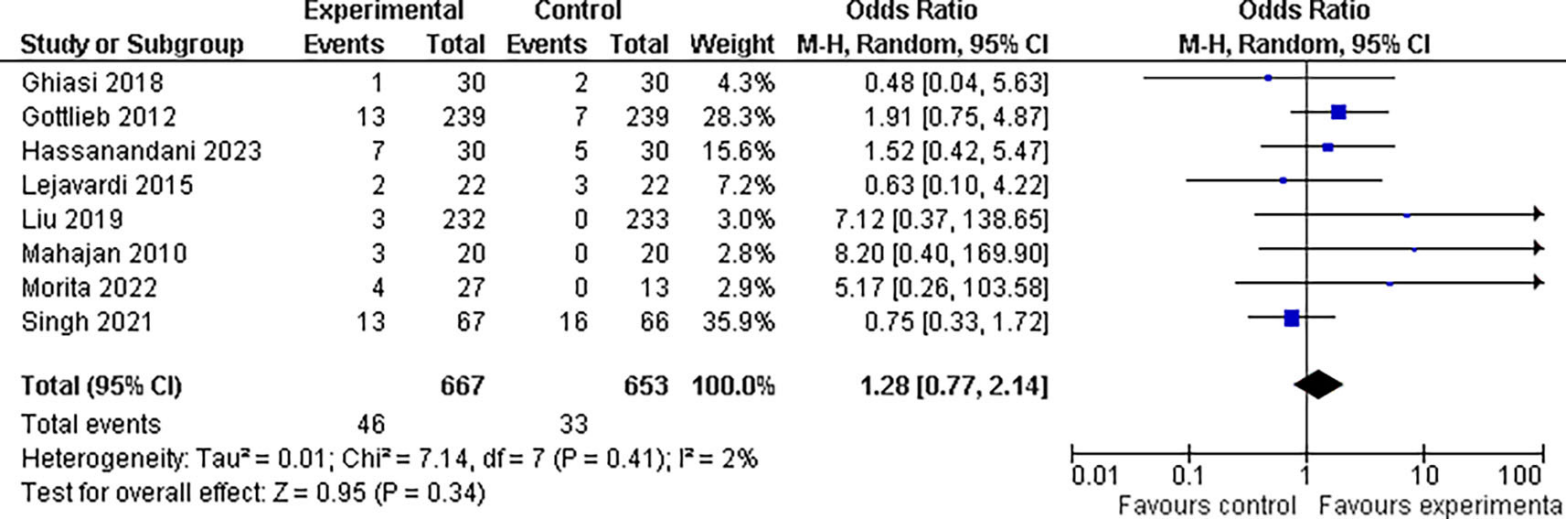
Forest Plot of Nausea occurrence in Combination Therapy verses control therapy patients with Plaque Psoriasis.

In our investigation, we found that the experimental group had a slightly higher incidence of nausea (6.9%, 46 of 667 patients) than the control group (5.0%, 33 of 653). However, the odds ratio of 1.28, with a 95% confidence interval of 0.77 to 2.14, and a p-value greater than 0.05, which is above the customary threshold of 0.05 for statistical significance, indicate that this difference is not statistically significant. As a result, our findings are insufficient to establish that the incidence of nausea differed considerably between the two treatment groups. This data highlights the need for more research to identify whether experimental treatment is related to a higher incidence of nausea than control treatment (refer Figure 9).

**3.5.3 Headache findings**

**Figure 10.**
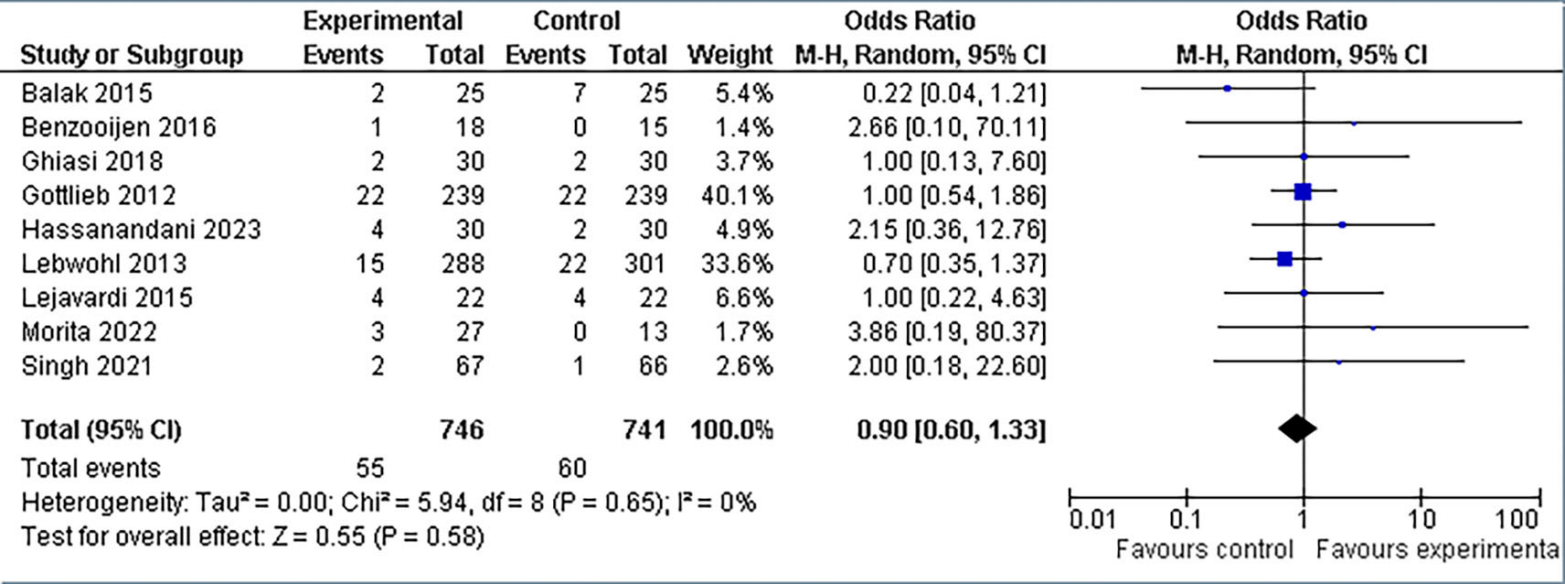
Forest Plot of Headache occurrence in Combination Therapy verses control therapy patients with Plaque Psoriasis.

The incidence of headache appeared to be equivalent between the experimental and control groups. In the experimental group of 746 patients, 7.4% (n = 55) complained of headaches, whereas in the control group of 741 patients, 8.1% (n = 60) reported comparable events. Overall, there appeared to be no statistically significant difference in the frequency of headaches between the combination therapy and control groups (refer Figure 10). This is seen by the overall odds ratio (OR) of 0.90 (95% CI: 0.60, 1.33), which is very near to 1.0. Furthermore, the p-value for the test of the overall effect is 0.58, which is significantly higher than the widely accepted significance level of 0.05. Furthermore, the heterogeneity statistic was noteworthy at (I ^2^ = 0%).

**3.5.4 Fatigue findings**

**Figure 11.**
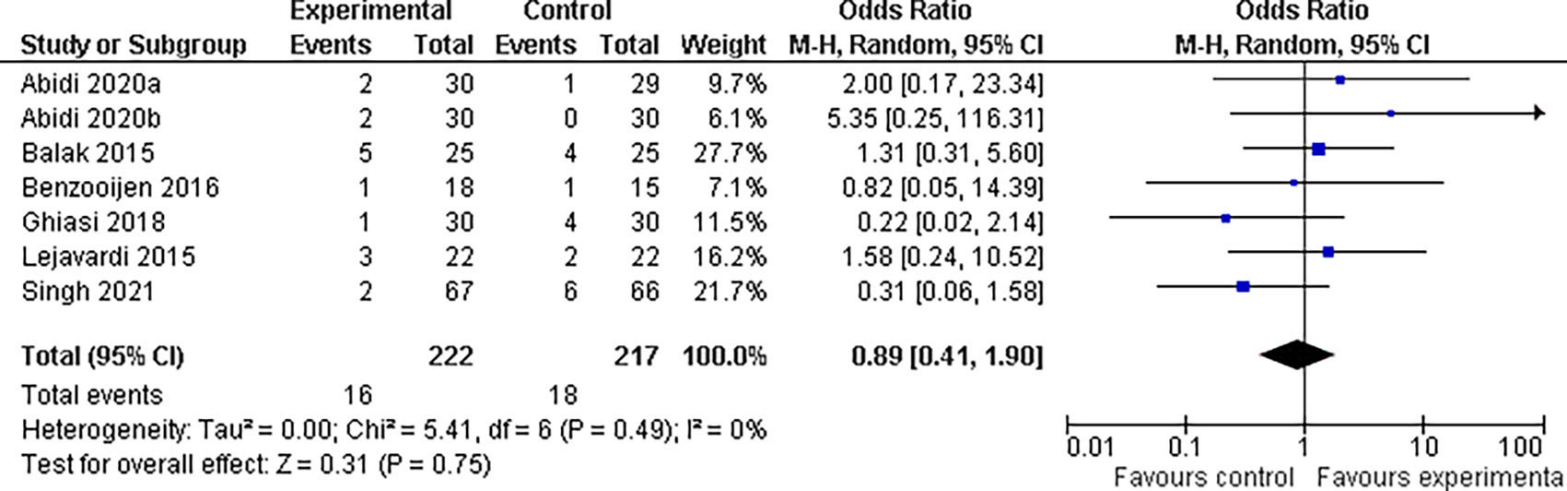
Forest Plot of Fatigue occurrence in Combination Therapy verses control therapy patients with Plaque Psoriasis.

Our study included eight trials that examined the incidence of fatigue with different medication combinations for the treatment of plaque psoriasis. The results revealed that the experimental group experienced considerably less fatigue than the control group did. The odds ratio for fatigue was 0.89 (95% confidence interval, 0.41 to 1.90), indicating that patients in the experimental group were 11% less likely to feel weariness than patients in the control group. This difference was not statistically significant (P = 0.75). There was no significant heterogeneity among the studies (I ^2^ = 0%), indicating consistent results across all the investigations (refer Figure 11).

## 4. Discussions

To study the efficacy and safety of combination therapy for plaque psoriasis in comparison with controls and other combination medications, we used a network meta-analysis approach. The lack of research articles directly comparing different combination therapies to placebo or to each other has led to the selection of this approach. According to prior systematic reviews and meta-analyses, combination therapies are either more effective than control groups or as effective. ^21^ Subjects aged 18 years or older were included in the study. Pregnant women and those with other genetic abnormalities were excluded. Only three large-scale, carefully planned clinical trials been conducted in the field of combination therapy have, ^8^^,^^20^^,^^22^ despite the fact that several RCTs involving patients have been conducted. Combination therapy showed a substantial improvement in PASI 75 response when compared to controls (OR: 2.53, P < 0.00001). Combination therapy that included pioglitazone showed the best correlation with beneficial outcomes (OR, 4.92; P = 0.0001).There was a substantial PASI 75 response also noted here, in the pioglitazone combination subgroup when compared with the placebo (OR = 4.64, 95% CI 2.03–10.60, P < .00001). ^23^ Therefore these results were consistent with their findings. Similar findings were obtained in ^24^ a study examining the effects of insulin sensitizers (metformin and pioglitazone) in patients with metabolic syndrome and psoriasis. Both the metformin and pioglitazone cohorts showed a significant improvement in Psoriasis Area and Severity Index (PASI) scores compared to the placebo group. Our subgroup analysis indicated substantial heterogeneity (I ^2^ = 34%); however, no significant differences were observed, indicating the need for more research with larger datasets. Promising trends were also observed for combinations such as methotrexate (OR, 2.56; 95% CI 1.67 – 3.94, P < 0.0001). A few additional combinations also produced good results, but the available data were insufficient to investigate them further, and this warrants further research with larger datasets. We assessed the frequency of particular adverse events (AEs) associated with the therapies under study to conduct a safety analysis. Trials that reported headache, nausea, exhaustion, and elevated or abnormal liver enzyme values were included. The proportion of individuals in a treatment arm who experienced each adverse event (AE) was our definition of “incidence.” Results are as follows: increase/abnormal levels of liver enzymes (OR: 1.89, 95% CI 0.69-5.22, P = 0.22), nausea (OR: 1.28, 95% CI 0.77-2.14, P = 0.34), fatigue (OR: O.89, 95% CI 0.41-1.90, P = 0.75), headache (OR: 0.90, 95% CI 0.60-1.33, P = 0.58). According to our investigation, there were no discernible differences in the occurrence of headaches, nausea, or abnormal liver enzyme levels between the groups. With 7% of the participants in the combination group and 8% in the control group, fatigue data indicated a possible lower risk in the combination group; however, this difference was not statistically significant. The incidence of SAEs was extremely low, and the overall safety profiles for combination regimes appeared manageable in the near term. Our study provides compelling evidence that treating plaque psoriasis with a combination of medications may be more successful than using them separately. This suggests that the effectiveness of various medication combinations for the treatment of plaque psoriasis may vary. Our findings may have implications in clinical practice. The observed variations in the safety and efficacy profiles across combination treatments indicate the potential for customized treatment regimens based on unique patient responses and features. However, before it can be widely used in clinical settings, further investigation is required to validate these results in more extensive, long-term investigations. It is critical to recognize some of the limitations of our meta-analysis. First, because there are few publications that directly compare therapies to a placebo, we used a network meta-analysis methodology in our approach. Compared with direct head-to-head comparisons, this approach naturally adds a degree of uncertainty, even though it permits indirect comparisons between treatments. Second, sample sizes varied among the included studies. A few studies have provided information from smaller sample sizes, which might have affected the reliability and applicability of our conclusions. Therefore, further investigation is required to validate these results for particular combinations and to fully evaluate safety profiles using larger datasets.

## 5. Conclusion

In summary, this network meta-analysis presents strong evidence that combination therapy is a potentially more effective treatment than monotherapy for plaque psoriasis. Although the pioglitazone combination seems to work well, more research is required to fully understand the potential of different combinations. The observed differences in the safety and efficacy profiles indicate the potential for individualized treatment plans based on the requirements of each patient. Further large-scale, long-term studies that directly compare various combination medications are essential to verify these results and to enhance patients’ treatment plans for plaque psoriasis.

## Data Availability

No Data are associated with this article. • Figshare: Efficacy and safety of various drug combinations of various drug combinations in treating plaque psoriasis: A meta analysis - Figures (Forest plots, flowcharts, RoB2). https://doi.org/10.6084/m9.figshare.25656471.v1 ^26^
• Supplementary material 1, Figshare: String operations employed in Efficacy and safety of various drug combinations in treating plaque psoriasis: A meta-analysis. https://doi.org/10.6084/m9.figshare.25656465.v1 ^27^
• Supplementary material 2, Figshare: Risk of bias excel tool with MACROS for included studies in ‘Efficacy and safety of various drug combinations in treating plaque psoriasis: A meta-analysis’. https://doi.org/10.6084/m9.figshare.25656411.v2 ^28^
• Supplementary material 3, Figshare: intervalidation META ANALYSIS.xlsx. https://doi.org/10.6084/m9. figshare.25656405.v2 ^29^ Data are available under the terms of the Creative Commons Attribution 4.0 International (CC-BY 4.0). Figshare: PRISMA CHECKLIST v2020 for ‘Efficacy and safety of various drug combinations in treating plaque psoriasis: A meta-analysis’. https://doi.org/10.6084/m9.figshare.25656444.v3 ^30^ Data are available under the terms of the Creative Commons Attribution 4.0 International (CC-BY 4.0).

## Software availability

• We used Review Manager software (version 5.4.1) to analyze the data; Note: Version 5.4.1 is discontinued, the current web version is available at https://training.cochrane.org/online-learning/core-software/revman
• Mendeley v1.19.8 and referencing software - https://www.mendeley.com/download-reference-manager/windows, https://www.mendeley.com/autoupdates/installers/preview
• WebPlotDigitizer v4.7- https://apps.automeris.io/wpd/
• Python Plotly v5.18.0 - https://chart-studio.plotly.com/create/
• Risk of Bias 2 assessment tool - https://www.riskofbias.info/welcome/rob-2-0-tool/current-version-of-rob-2
