# Supplementary material for: Efficacy and safety of various drug combinations in treating plaque Psoriasis: A meta-analysis
Source: F1000Res. 2025 Jan 28;13:453. Originally published 2024 May 7. [Version 2] doi: 10.12688/f1000research.149172.2 (PMC11803402; doi:10.12688/f1000research.149172.2)
Supplement: Peer review history

# Reviewer report: Reviewer response for version 2

**DOI:** 10.5256/f1000research.177366.r363099

**Published:** 2025-02-06

**Authors:** Naoko Kanda

**Affiliations:** 1 Nippon Medical School Chiba Hokusoh Hospital, Inzai, Japan

The authors well revised the article and improved the contents.

Are the rationale for, and objectives of, the Systematic Review clearly stated?

Partly

Is the statistical analysis and its interpretation appropriate?

I cannot comment. A qualified statistician is required.

If this is a Living Systematic Review, is the ‘living’ method appropriate and is the search schedule clearly defined and justified? (‘Living Systematic Review’ or a variation of this term should be included in the title.)

Not applicable

Are sufficient details of the methods and analysis provided to allow replication by others?

Partly

Are the conclusions drawn adequately supported by the results presented in the review?

Partly

Reviewer Expertise:

Cutaneous immunology, atopic dermatitis, psoriasis

I confirm that I have read this submission and believe that I have an appropriate level of expertise to confirm that it is of an acceptable scientific standard.

# Reviewer report: Reviewer response for version 1

**DOI:** 10.5256/f1000research.163592.r281705

**Published:** 2024-06-17

**Authors:** Romana Čeović

**Affiliations:** 1 University of Zagreb, Zagreb, Croatia

The article is nicely written and educational as it provides detailed information on the safety and effectiveness of various combination therapies used in the treatment of psoriasis. Statistical processing accurately displays the data that is most important in daily praxis.  Although the pioglitazone combination seems to work well, more research is required to fully understand the potential of different combinations. Further large-scale, long-term studies that directly compare various combination medications are essential to verify these results and to enhance patients’ treatment plans for plaque psoriasis.

Are the rationale for, and objectives of, the Systematic Review clearly stated?

Yes

Is the statistical analysis and its interpretation appropriate?

Yes

If this is a Living Systematic Review, is the ‘living’ method appropriate and is the search schedule clearly defined and justified? (‘Living Systematic Review’ or a variation of this term should be included in the title.)

Yes

Are sufficient details of the methods and analysis provided to allow replication by others?

Yes

Are the conclusions drawn adequately supported by the results presented in the review?

Yes

Reviewer Expertise:

Psoriasis and erithematosquamous dermatosis, dermatological oncology.

I confirm that I have read this submission and believe that I have an appropriate level of expertise to confirm that it is of an acceptable scientific standard.

# Reviewer report: Reviewer response for version 1

**DOI:** 10.5256/f1000research.163592.r275508

**Published:** 2024-05-14

**Authors:** Naoko Kanda

**Affiliations:** 1 Nippon Medical School Chiba Hokusoh Hospital, Inzai, Japan

1.The term ‘ combination therapy’ appears to mean the combination of more than one systemic treatments, and does not appear to include combination of systemic and topical treatments. However, the combination of topical and systemic treatments is included in this study.

 Such analysis is rather queer since such combination is ordinarily done in real-world clinical practice.

 2.The choice of drugs is also very much biased: pioglitazone, methotrexate, apremilast, etanercept, calcipotriol, betamethasone, cyclosporine, clobetasol propionate, acitretin, fumarate, and cetirizine.

 Among these, calcipotriol, betamethasone, clobetasol propionate are topical treatments and should be deleted from this review.

 Cetilizine is a second-generation antihistamine while pioglitazone is an anti-diabetic medication. The reasons for choosing these agents for psoriasis are unknown. Etenercept is the only biologic among the agents, and thus dealing with this drug in parallel to other small molecule systemic agents should be avoided.

 3.The definition of palmoplantar psoriasis is ambiguous. Is that palmo-plantar pustulosis or psoriasis appearing on palmoplantar areas?

Are the rationale for, and objectives of, the Systematic Review clearly stated?

Partly

Is the statistical analysis and its interpretation appropriate?

I cannot comment. A qualified statistician is required.

If this is a Living Systematic Review, is the ‘living’ method appropriate and is the search schedule clearly defined and justified? (‘Living Systematic Review’ or a variation of this term should be included in the title.)

Not applicable

Are sufficient details of the methods and analysis provided to allow replication by others?

Partly

Are the conclusions drawn adequately supported by the results presented in the review?

Partly

Reviewer Expertise:

Cutaneous immunology, atopic dermatitis, psoriasis

I confirm that I have read this submission and believe that I have an appropriate level of expertise to state that I do not consider it to be of an acceptable scientific standard, for reasons outlined above.

# Response

**Published:** 2024-05-14

**Authors:** Snehasis Nayak

**Affiliations:** Medicine, Not Applicable, Bengaluru, Karnataka, India

Firstly, thank you ma'am for your valuable insight about our paper. We do appreciate your patience and time for reviewing our article. But I would like to address certain points of comment on my article.

 Comment 1 - 

 We acknowledge that the term can sometimes be interpreted more narrowly to refer solely to systemic combinations.

 However, in this study, we deliberately adopted a broader definition of combination therapy to encompass all possible treatment combinations, including those that involve both systemic and topical medications. This approach aligns with real-world clinical practice, where dermatologists frequently combine topical and systemic therapies to achieve optimal results for patients with plaque psoriasis. This includes the synergistic effects of combining topical and systemic therapies, which is a common and effective approach in real-world clinical practice. While there is extensive study on the efficacy and safety of combination therapies compared to placebo or monotherapy, a comparative analysis between various combination therapies remains relatively unexplored in recent times.

 We aimed to address this specific gap by investigating the relative efficacy and safety profiles of different combination regimens for plaque psoriasis.

 Comment 2 - 

 ​​It's important to clarify that our choice of drugs was not biased, but rather based on established evidence from several randomized controlled trials (RCTs) regarding their efficacy in treating plaque psoriasis. Pioglitazone, for instance, has been studied extensively in the context of psoriasis treatment, with numerous RCTs such as Ghiasi 2020, VG Hafiz 2015, and Lajevardi 2015 demonstrating its effectiveness.

 Moreover, our study is a meta-analysis, meaning that we synthesized data from existing literature rather than arbitrarily selecting treatments. We included a range of systemic and topical treatments to provide a comprehensive overview of available options for psoriasis management. Therefore, the inclusion of calcipotriol, betamethasone, and clobetasol propionate, which are topical treatments, is justified as they are commonly used in clinical practice. Comparing biologics with small molecule treatments is crucial for understanding their relative efficacy and safety. This comparison helps clinicians make informed choices about integrating biologics into treatment regimens. Including a biologic like etanercept ensures our study covers all major classes of psoriasis treatments, providing a complete picture of therapeutic options. We have also emphasized on the need of further research into these areas as well. We have not stated that these could be used as definitive treatment regimes but instead as possible treatment regimes.

 Thus, our approach was systematic and evidence-based, aiming to provide clinicians with comprehensive information to guide treatment decisions for psoriasis patient. Furthermore, it's important to acknowledge that this study utilized a network meta-analysis approach, allowing for indirect comparisons between treatments. We have highlighted the limitations of this methodology in our paper to ensure transparency and help readers interpret the findings appropriately 

 Comment 3 -

 I see where the confusion might arise. While palmoplantar psoriasis shares similarities with plaque psoriasis in terms of its underlying condition, it's essential to differentiate them as palmoplantar psoriasis specifically. I would make the necessary correction related to that.

 We hope these points clarify the rationale behind our study design and drug selection; and we appreciate your consideration of our perspective.

 Best regards,

 Snehasis Nayak
